# Causal Influences of Micronutrients on Anxiety: Insights From an Observational and Mendelian Randomization Analysis

**DOI:** 10.1002/fsn3.71166

**Published:** 2025-11-05

**Authors:** Xinyu Fang, Qian Zhao, Peizi Liu, Ancha Baranova, Hongbao Cao, Xiangrong Zhang, Fuquan Zhang

**Affiliations:** ^1^ Department of Geriatric Psychiatry The Affiliated Brain Hospital of Nanjing Medical University Nanjing China; ^2^ Department of Psychiatry The Affiliated Brain Hospital of Nanjing Medical University Nanjing China; ^3^ School of Systems Biology George Mason University Fairfax USA; ^4^ Research Centre for Medical Genetics Moscow Russia; ^5^ Institute of Neuropsychiatry The Affiliated Brain Hospital of Nanjing Medical University Nanjing China

**Keywords:** anxiety, mendelian randomization, micronutrients, minerals, vitamins

## Abstract

An increasing number of studies have begun to focus on the role of micronutrients (vitamins and minerals) in mental disorders, including anxiety. Previous observational and clinical studies have suggested a relationship between micronutrients and anxiety, yet the causal link between them remains unclear. We aimed to detect the potential causal relationships between genetically predicted supplementation with vitamins (including vitamins A, B, B6, B12, C, D, E, folate, and multivitamins), minerals (including calcium, iron, magnesium, selenium, and zinc), and anxiety through Mendelian randomization (MR) analysis, the Bayesian colocalization analysis, and real‐world observational data. Summary genome‐wide association study datasets covering various supplements from the UK Biobank (*N* = 64,979 ~ 462,933) and anxiety (*N* = 346,542) from the FinnGen project R10 were utilized in our MR analyses. Then, the Bayesian colocalization analysis was used to validate the MR findings. Finally, we analyzed the association between micronutrients and anxiety by mining the United States Food and Drug Administration Adverse Event Reporting System (FAERS) database (2004Q1 ~ 2024Q2). IVW‐based MR study revealed the significant protective effects of vitamin C (OR: 0.38, *p* = 0.032), magnesium (OR: 0.15, *p* = 0.035), and selenium (OR: 0.03, *p* = 0.020) against anxiety disorders. However, the colocalization analysis did not provide any evidence of shared causal variants between supplementation with vitamin C, magnesium, selenium, and anxiety disorders. Analysis of the FAERS database showed that vitamin C (ROR: 0.30, 95% CI: 0.16–0.59, *p* = 0.0002) and magnesium use (ROR: 0.32, 95% CI: 0.18–0.57, *p* < 0.0001) were associated with decreased risks of anxiety; while no cases of anxiety were reported for selenium use. Our study provides suggestive genetic evidence of the causal protective role of vitamin C, magnesium, and selenium on the risk of anxiety and supports targeting anxiety phenotypes with nutritional interventions.

## Graphical abstract

Overview of study design. FAERS, the United States Food and Drug Administration Adverse Event Reporting System; GWAS, genome‐wide association study; IVs, instrumental variables; IVW, inverse‐variance weighted; MR, Mendelian randomisation; SNPs, single‐nucleotide polymorphisms; WM, weighted median.

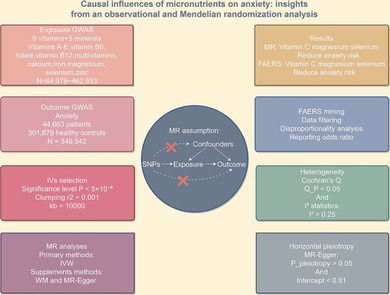

**Abbreviations**
FAERS: The United States Food and Drug Administration Adverse Event Reporting System
GWAS: genome‐wide association studies
IVs: instrumental variables
IVW: inverse variance weighted
MR: Mendelian randomization
PT: preferred item
ROR: reporting odds ratio
SNPs: single‐nucleotide polymorphisms
TSMR: two‐sample Mendelian randomization
WM: weighted median

## 1 Background

Anxiety disorders are prevalent globally (Buist‐Bouwman et al. 2006). According to the latest epidemiological data collected in China, the incidence rate of anxiety disorders ranks first among mental disorders, with lifetime prevalence and 12‐month prevalence reaching as high as 7.6% (Huang et al. 2019). These disorders are characterized by excessive worry, fear, or unease (Morrison et al. 2024; Pang et al. 2024), and are usually combined with significant distress or impairment in personal, occupational, and other areas of functioning; therefore, imposing a substantial burden on the families and the whole society (Chen et al. 2024; Liu, Yang, et al. 2024; Cheng et al. 2024). Although the pathogenesis of anxiety is multifactorial, involving a complex interplay of genetic (Pan et al. 2024; Li et al. 2023), environmental, and lifestyle factors (Yu et al. 2024), there has been a growing interest in the role of micronutrients and their deficiencies as possible risk factors.

Vitamins and the minerals that work in concert with vitamins are important nutritional components regulating various functions within the human body. Their steady‐state and augmented levels have been associated with the pathogenesis of various mood disorders, including anxiety disorders (Hu et al. 2024; Nakamura et al. 2019). A substantial body of evidence indicates that vitamins may modulate the intensity of emotions (Xiao et al. 2021), for example, by modifying the levels of serotonin (Ceolin et al. 2021) and the activity of tyrosine hydroxylase (Saeedfar et al. 2023). In addition, the receptors for vitamins are abundant in brain regions related to anxiety and depression (Menon et al. 2020). Similarly, minerals have been found to influence the expression of some specific molecules and also the overall functionality of the neural pathways implicated in mood regulation (Siuciak et al. 2007).

Observational studies have found that inadequate intake or deficiency of certain vitamins, such as vitamin D or group B vitamins, correlates with higher prevalence and severity of anxiety symptoms (Domacassé et al. 2024; Al Jassem et al. 2024). Moreover, both animal experiments and clinical studies demonstrate that a higher intake of these vitamins through diet or exogenous supplementation contributes to alleviating the intensity of anxiety symptoms and reducing the frequency of anxiety attacks (Taiwo et al. 2024; Arabshahi et al. 2024). On the other hand, increased anxiety and stress responses have been linked to deficiencies in some minerals, while adequate intake of iron and zinc aids in reducing these symptoms (Młyniec et al. 2017; Muscaritoli 2021). Here is, however, a certain lack of causal evidence for a connection between supplementation with micronutrients and anxiety phenotypes, which is due to the observational nature of a majority of the studies for such associations.

Indeed, traditional observational research has many limitations, which include the presence of confounding biases and the potential for reverse causation (Flegal et al. 2011). These limitations may be solved using Mendelian Randomization (MR), an analytical approach that employs genetic variants as instrumental variables (IVs) to derive causal inferences, which are impossible to solve in observational studies. This method capitalizes on the random allocation of genetic variations at conception, assuming that these variants are linked to modifiable risk factors (such as micronutrient intake) and corresponding outcomes (such as anxiety) exclusively through the risk factor, rather than through any confounding influences. MR has become a widely adopted approach for uncovering potential causal determinants of human disease (Fu et al. 2024; Liu, Baranova, and Zhang 2024; Baranova et al. 2023). Recently, MR analysis has been used for establishing potential causal relationships between micronutrients and mental disorders, including schizophrenia, bipolar disorder, and depression (Wu et al. 2024; Guo et al. 2023), while few reports were made on connections between supplementation with vitamins and minerals and anxiety disorders.

In the current study, we employed a two‐sample MR design to investigate the potential causal links between genetic signatures underlining supplementation with various micronutrients, including vitamins (vitamins A, B, B6, B12, C, D, E, folate, and multivitamins), minerals (including calcium, iron, magnesium, selenium, and zinc), and the risk of anxiety disorders. Then, the Bayesian colocalization analysis was used to validate the MR findings. Additionally, to further corroborate these MR findings, we analyzed real‐world observational data from the United States Food and Drug Administration Adverse Event Reporting System (FAERS) database (2004Q1–2024Q2); thus, extracting complementary evidence for the micronutrient–anxiety relationships. The objective of our research was to provide more definitive evidence on whether micronutrient supplementation could aid in an effective intervention for either the prevention or treatment of anxiety disorders.

## 2 Methods

### 2.1 Study Design and Data Sources

In the present study, we conducted a two‐sample MR study to examine the potential causal relationship between supplementation with various micronutrients and anxiety disorders. In MR, three assumptions were made about IVs: they must be closely linked to the exposure, not related to known confounders, and influence the outcome solely through their effect on the exposure (Lawlor 2016).

Our MR study leveraged the publicly accessible genome‐wide association study (GWAS) databases, known for their comprehensive coverage and capacity to detect genetic variants linked to complex traits or diseases, all of which have been ethically approved and consented to. The GWAS data for anxiety disorders were obtained from the FinnGen project R10 (https://storage.googleapis.com/finngen‐public‐data‐r10/summary_stats/finngen_R10_F5_ALLANXIOUS.gz), comprising 44,663 patients with anxiety disorders and 301,879 healthy controls (*N* = 346,542) (Kurki et al. 2023). The pooled data for the supplementation with 14 micronutrients were obtained from the IEU OpenGWAS Project (IEU), including that for vitamin A (*N* = 460,351) (https://gwas.mrcieu.ac.uk/datasets/ukb‐b‐9596), vitamin B (*N* = 460,351) (https://gwas.mrcieu.ac.uk/datasets/ukb‐b‐10188), vitamin B6 (*N* = 64,979) (https://gwas.mrcieu.ac.uk/datasets/ukb‐b‐129), folate (*N* = 460,351) (https://gwas.mrcieu.ac.uk/datasets/ukb‐b‐3563), vitamin B12 (*N* = 462,933) (https://gwas.mrcieu.ac.uk/datasets/ukb‐b‐18819), vitamin C (*N* = 460,351) (https://gwas.mrcieu.ac.uk/datasets/ukb‐b‐15175), vitamin D (*N* = 460,351) (https://gwas.mrcieu.ac.uk/datasets/ukb‐b‐12648), vitamin E (*N* = 460,351) (https://gwas.mrcieu.ac.uk/datasets/ukb‐b‐12506), multivitamins (*N* = 460,351) (https://gwas.mrcieu.ac.uk/datasets/ukb‐b‐16812), calcium (*N* = 461,384) (https://gwas.mrcieu.ac.uk/datasets/ukb‐b‐7043), iron (*N* = 461,384) (https://gwas.mrcieu.ac.uk/datasets/ukb‐b‐14863), magnesium (*N* = 64,979) (https://gwas.mrcieu.ac.uk/datasets/ukb‐b‐5536), selenium (*N* = 461,384) (https://gwas.mrcieu.ac.uk/datasets/ukb‐b‐19158), and zinc (*N* = 461,384) (https://gwas.mrcieu.ac.uk/datasets/ukb‐b‐13891). This study adheres to the STROBE‐MR guidelines. For this analysis, no additional ethical or informed consent was required.

### 2.2 Selection Criteria for Genetic Variants

IVs were selected according to the following criteria: single nucleotide polymorphisms (SNPs) with a genome‐wide significance level of *p* < 5 × 10^−8^ and a clumping algorithm with a cutoff of *r* ^2^ = 0.001 and kb = 10,000 were used to avoid linkage disequilibrium (LD). Finally, a total of 20 SNPs for vitamin A, 33 SNPs for vitamin B, 6 SNPs for vitamin B6, 19 SNPs for folate, 8 SNPs for vitamin B12, 42 SNPs for vitamin C, 27 SNPs for vitamin D, 24 SNPs for vitamin E, 73 SNPs for multivitamins, 42 SNPs for calcium, 31 SNPs for iron, 7 SNPs for magnesium, 16 SNPs for selenium, and 37 SNPs for zinc were selected for MR analyses.

### 2.3 Statistical Analysis

We employed various MR approaches to estimate the potential bidirectional causal associations between micronutrients and anxiety, including inverse variance weighted (IVW), weighted median (WM), and MR‐Egger techniques. IVW was utilized as the primary approach, and others as supplements. For sensitivity analysis, we first performed Cochran's *Q* and *I* ^2^ statistics to gauge the extent of heterogeneity (both *I* ^2^ > 0.25 and *p* < 0.05). We also assessed horizontal pleiotropy with P_pleiotropy > 0.05 and MR‐Egger intercept < 0.01 (Fu et al. 2024; Baranova et al. 2024). In IVW, the statistical tests were two‐sided, with a *p* < 0.05 considered as suggestive evidence for a potential causal association. Corrections for multiple comparisons were also conducted using the false discovery rate (FDR). All analyses were performed in R (version 4.3.1).

### 2.4 Colocalization Analysis

Utilizing the “coloc” package in R, we then conducted Bayesian colocalization analysis to explore the potential shared genetic causal variants between detected micronutrients in MR analysis and anxiety within a designated genomic region. For this purpose, we established a 500 kb window centered on the lead SNP to pinpoint pertinent genomic areas. The SNPs were extracted and mapped to genes using the online SNPnexus database (https://www.snp‐nexus.org/v4/). According to the established guidelines, a posterior probability of H4 (PP.H4) greater than 0.80 is indicative of robust evidence for colocalization.

### 2.5 Anxiety Adverse Event Signal Mining

Adverse event data were obtained from the United States Food and Drug Administration Adverse Event Reporting System (FAERS) database, which was analyzed with an online tool “FastSignal” (http://www.faers.trit‐bio.com/). FAERS is a large public database of adverse events spontaneously reported by healthcare workers, pharmacists, and consumers worldwide. To detect the presence or absence of adverse event signals, we selected vitamin C, magnesium, or selenium as the target drugs, and anxiety as the target adverse event. The data used in this study covered Q1 2004 to Q2 2024. In the present study, we collected variables that are not limited to the patient demographics (such as age and sex), reporting regions, and severity of the adverse event. After excluding duplicates and outliers based on the data cleaning criteria of the FAERS, a total of 54,336,884 reports were finally retained. To compare the risk of adverse events between the drug of interest (vitamin C, magnesium, or selenium) and other drugs in the database, a two‐by‐two contingency table was constructed and a disproportionality analysis (DPA) was executed using the reporting odds ratio (ROR), using the formula: ROR = (N1 × N4)/(N2 × N3), where N1 indicates the number of times the target drug led to the target adverse event; N2 represents the number of other adverse events caused by the target drug; N3 indicates the number of times other drugs caused the target adverse event; N4 represents the number of other drugs causing other adverse events. The 95% confidence intervals (CIs) were calculated. A lower 95% CI for ROR value > 1 and the number of cases (N) ≥ 3 indicates an association between the drug and the adverse event (Sakaeda et al. 2013).

## 3 Results

### 3.1 MR Analyses

**FIGURE 1:**
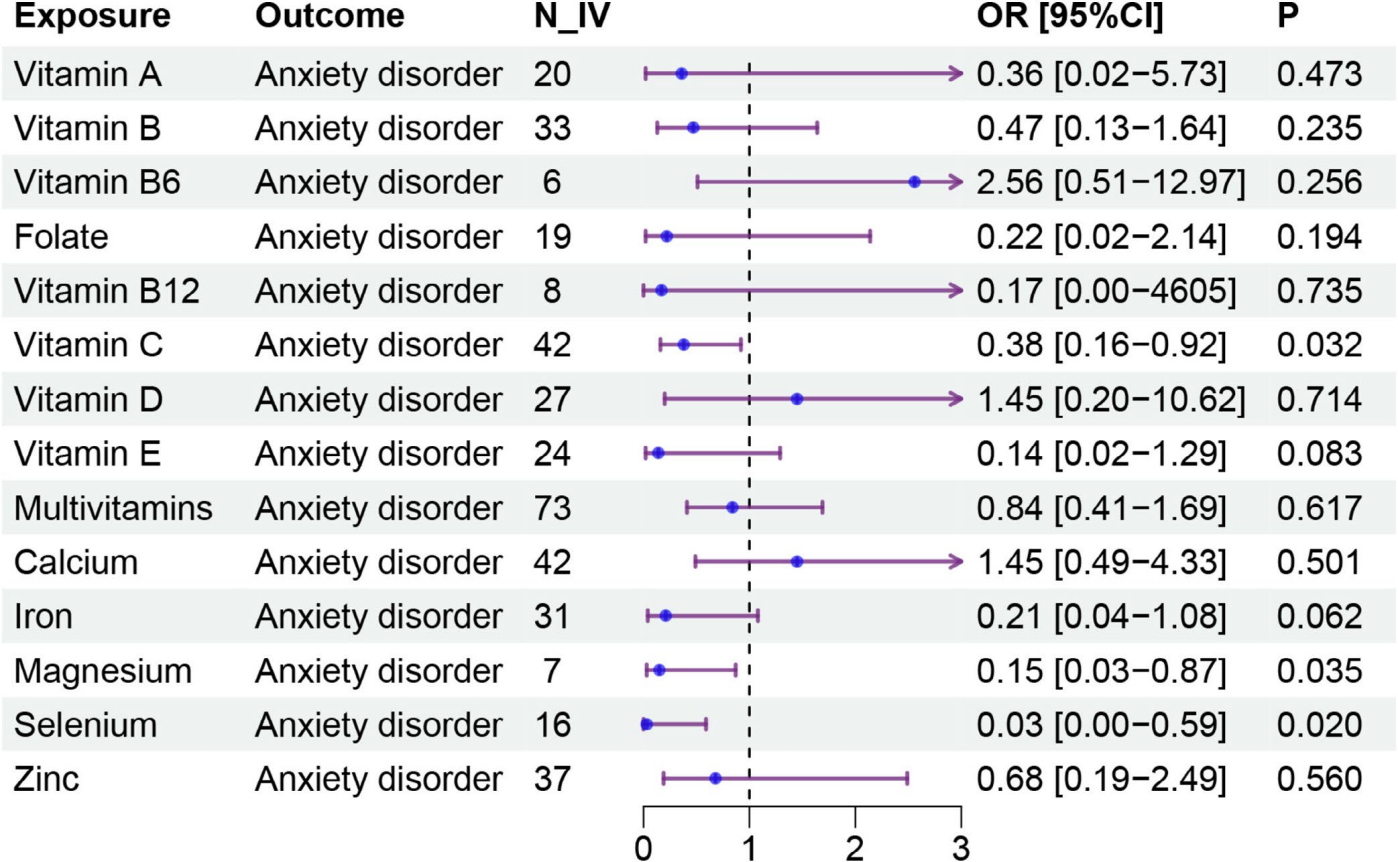
Causal effects of micronutrient intake on anxiety disorder using the IVW method. Forest plot illustrating the causal effect of micronutrient supplementation on anxiety disorder. Each row represents the odds ratio (OR) with corresponding 95% confidence intervals (CI) for a specific micronutrient. IVW represents inverse‐variance weighted. N‐IV represents the number of instrumental variables used for each analysis.

**FIGURE 2:**
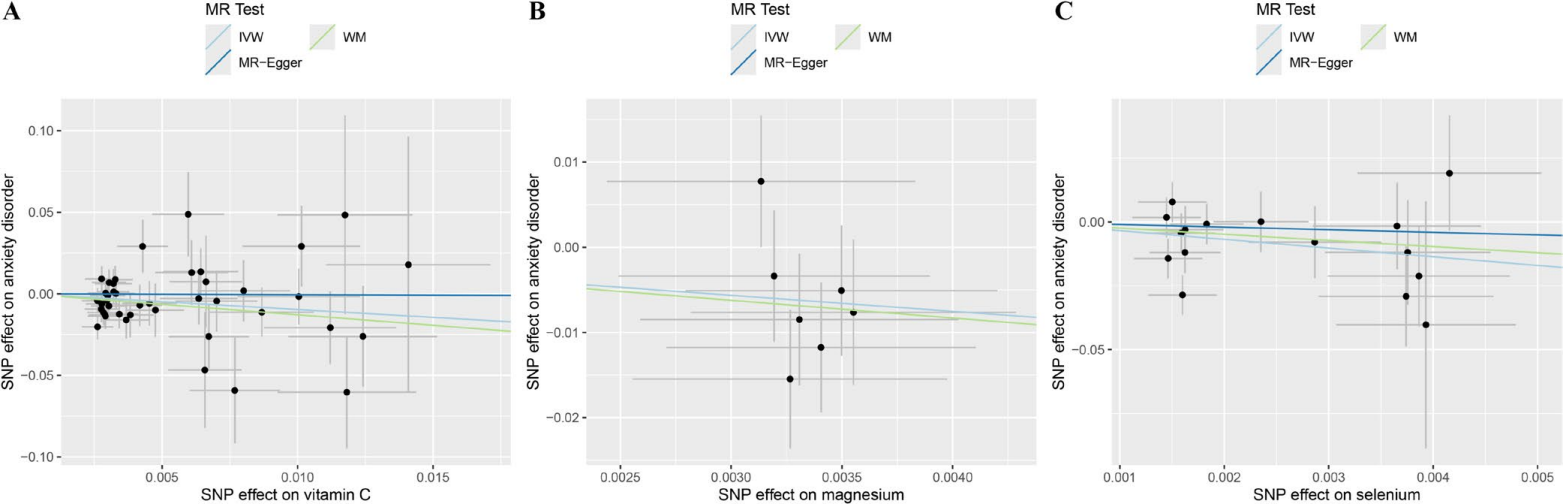
Scatter plot of causal effects of micronutrient intake on anxiety disorder. Scatter plots show Mendelian randomization (MR) analysis of the effect of genetic variants (SNPs) associated with different micronutrient intakes on anxiety disorder. (A) Vitamin C. (B) Magnesium. (C) Selenium. SNP effects are plotted on the *x*‐axis and corresponding anxiety effects are on the *y*‐axis. The three MR methods used are inverse‐variance weighted (IVW, blue), weighted median (WM, green), and MR‐Egger (black), with their regression lines overlaid. Error bars indicate the standard error of SNP effects.

Table 1, Figures 1 and 2, and Figure S1 show the results of two‐sample MR analyses exploring the relationship between micronutrient levels and the risk for anxiety disorders. Analysis showed that the genetic signatures underlying the supplementation with vitamin C (IVW method: OR: 0.38, 95% CI: 0.16–0.92, *p* = 0.032), magnesium (IVW method: OR: 0.15, 95% CI: 0.03–0.87, *p* = 0.035), and selenium (IVW method: OR: 0.03, 95% CI: 0.00–0.59, *p* = 0.020) are protective against anxiety disorders. However, no significant connections with the risks of anxiety were noted for genetic signatures influencing the supplementation with vitamins A (OR: 0.36, 95% CI: 0.02–5.73, *p* = 0.473), B (OR: 0.47, 95% CI: 0.13–1.64, *p* = 0.235), B6 (OR: 2.56, 95% CI: 0.51–12.97, *p* = 0.256), B12 (OR: 0.17, 95% CI: 0.00–46.05, *p* = 0.735), D (OR: 1.45, 95% CI: 0.20–10.62, *p* = 0.714), folate (OR: 0.22, 95% CI: 0.02–2.14, *p* = 0.194), multivitamins (OR: 0.84, 95% CI: 0.41–1.69, *p* = 0.617), calcium (OR: 1.45, 95% CI: 0.49–4.33, *p* = 0.501), zinc (OR: 0.68, 95% CI: 0.19–2.49, *p* = 0.560). There was also a tendency for a possible causal relationship between the supplementation with vitamin E (OR: 0.14, 95% CI: 0.02–1.29, *p* = 0.083) and with iron (OR: 0.21, 95% CI: 0.04–1.08, *p* = 0.062) and anxiety risks. The causal effect estimates calculated through the WM and MR‐Egger methods showed consistency of the observed trends. After applying the FDR correction across all 12 micronutrient exposures, none of the associations for vitamin C, magnesium, or selenium remained significant (FDR corrected *p* > 0.05).

**TABLE 1:** Results of micronutrients intake on anxiety disorder returned by three different types of Mendelian randomization analyses.

Exposure	N_IV	Methods	OR [95% CI]	*p*
Vitamin A	20	IVW	0.36 [0.02–5.73]	0.473
Vitamin B	33	IVW	0.47 [0.13–1.64]	0.235
Vitamin B6	6	IVW	2.56 [0.51–12.97]	0.256
Folate	19	IVW	0.22 [0.02–2.14]	0.194
Vitamin B12	8	IVW	0.17 [0.00–4604.55]	0.735
Vitamin C	42	IVW	0.38 [0.16–0.92]	0.032
Vitamin D	24	IVW	1.45 [0.20–10.62]	0.714
Vitamin E	42	IVW	0.14 [0.02–1.29]	0.083
Multivitamins	73	IVW	0.84 [0.41–1.69]	0.617
Calcium	42	IVW	1.45 [0.49–4.33]	0.501
Iron	31	IVW	0.21 [0.04–1.08]	0.062
Magnesium	7	IVW	0.15 [0.03–0.87]	0.035
Selenium	16	IVW	0.03 [0.00–0.59]	0.020
Zinc	37	IVW	0.68 [0.19–2.49]	0.560

Abbreviations: CI, confidence interval; IVW, inverse variance weighted; N_IV, number of instrumental variables; OR, odds ratio; WM, weighted median.

For the sensitivity analysis, Cochrane's *Q* and *I* ^2^ tests were run, to indicate the absence of heterogeneity in most MR analyses, except for that related to vitamin D, multivitamins, and calcium in Cochrane's *Q* test, and that for folate, vitamin B12, vitamin D, vitamin E, multivitamins, calcium and magnesium in the *I* ^2^ test (Table S1). In addition, to evaluate the potential for horizontal pleiotropy and bias from ineffective IVs, we also calculated MR‐Egger intercepts. The results summarized in Table S1 show evidence for pleiotropy in causal effects of vitamin B6 (Egger_intercept = 0.031, P_pleiotropy = 0.739), vitamin B12 (Egger_intercept = 0.023, P_pleiotropy = 0.632), vitamin D (Egger_intercept = −0.011, P_pleiotropy = 0.095) and magnesium (Egger_intercept = 0.068, P_pleiotropy = 0.374), whereas no significant horizontal pleiotropy was observed for the other 10 micronutrients (All Egger_intercept < 0.01 and P_pleiotropy > 0.05).

### 3.2 Colocalization Analysis

There were no shared causal variants in the associations between supplementation with vitamin C, magnesium or selenium, and anxiety disorders (PP.H4 < 0.80). The values of PP.H4 across each region are presented in Table S2.

### 3.3 Anxiety Adverse Event Signal Mining

We used the FAERS database to detect possible adverse event signals (the results are displayed in Table 2). Data analysis showed that when the target drugs were vitamin C (ROR for anxiety: 0.30, 95% CI: 0.16–0.59, N1 = 9, *p* = 0.0002) and magnesium (ROR for anxiety: 0.32, 95% CI: 0.18–0.57, N1 = 12, *p* < 0.0001), a total of 6230 and 7810 adverse events were detected, respectively. Therefore, no adverse reaction signals related to anxiety were detected. When the target drug was selenium, no anxiety reports were returned (N1 = 0). We conclude that FAERS analyses supported MR findings that vitamin C, magnesium, and selenium supplementation decrease the risks of anxiety.

**TABLE 2:** Anxiety adverse events signals associated with micronutrient drugs.

Drug	N1	N2	N3	N4	*N*	ROR [95% CI]	*p*
Vitamin C	9	6221	256,787	54,073,867	54,336,884	0.30 [0.16–0.59]	0.0002
Magnesium	12	7798	256,784	54,072,290	54,336,884	0.32 [0.18–0.57]	< 0.0001

Abbreviations: CI, confidence interval; *N*, total number of adverse event cases; N1, number of times the target drug led to the target adverse event; N2, number of other adverse events caused by the target drug; N3, number of times other drugs caused the target adverse event; N4, number of other drugs causing other adverse events; ROR, reporting odds ratio.

## 4 Discussion

In this MR study, we aimed to investigate potential causal relationships between the genetic signatures contributing to voluntary or prescribed supplementation with 14 micronutrients and the risk of anxiety. Marked protective associations were revealed for supplementation with vitamin C, magnesium, and selenium. Additionally, a tendency toward significance was noted for causal relationships between supplementation with vitamin E and iron, and anxiety phenotypes.

These findings suggest that inherent or acquired deficiencies of these micronutrients may be involved in the pathogenesis of anxiety disorders and that supplementing their levels may alleviate anxiety symptoms and, possibly, prevent their onset. No such associations were found for supplementing with vitamin A, group B, B6, B12, D, folate, multivitamins, calcium, or zinc, and anxiety disorders.

Vitamins and minerals are essential micronutrients that play crucial roles in vital physiological processes within the human body, including the maintenance of a robust immune system and the nourishment of neuronal connections. Notably, the effects of the vitamins and minerals are commonly reported in diseases with hematological or neurological manifestations (O'Leary and Samman 2010), while anxiety lacks measurable somatic symptoms. Because of that, the relationships between micronutrient intake and anxiety have been overlooked, with a scarce set of correlational studies reporting inconsistent findings (Sahu et al. 2022; Mozaffari et al. 2021). The discrepancies in the results of these studies may have arisen from the inherent limitations of observational research, such as confounding biases and reverse causation (Flegal et al. 2011). With the advent of MR studies, a convenient and persuasive opportunity to reveal the potential causal relationships between micronutrients and psychological disorders has presented itself.

In light of the ability of MR to solve contradictions reported in observational research, our findings concerning the role of vitamin C are especially illustrative. Vitamin C deficiency is highly prevalent in the inpatient psychiatric setting (Bari et al. 2023). Nevertheless, some reports have shown that subjects with anxiety disorders have higher vitamin C levels when compared to healthy controls (Islam et al. 2014). However, in rats chronically exposed to tramadol, supplementation with vitamin C alleviated anxiety (Taiwo et al. 2024). In other animal studies, the anxiolytic effects of vitamin C were comparable to those of escitalopram (Gammoh et al. 2023). Nevertheless, the results of human studies aimed at the improvement of anxiety symptoms through supplementing with vitamin C were inconsistent. For example, vitamin C supplementation did reduce anxiety and depressive symptoms in students (de Oliveira et al. 2015), while no effects of vitamin C supplementation on anxiety were found in pregnant women (Carr et al. 2023). It is worth noting that the COVID‐19 pandemic has significantly increased the incidence of anxiety (Li et al. 2024), and an increase in vitamin C consumption, which was also noticeable during the COVID‐19 pandemic, has not helped as much as it may be expected (Kontopoulou et al. 2023). On the other hand, as a benchmark for COVID‐19 pandemics was never established, the beneficial effects of increased vitamin C consumption may have remained obscured by the sheer breadth of COVID‐19‐related effects.

In this MR study, overcoming the shortcomings of observational research, the protective effect of vitamin C on anxiety symptoms was confirmed. Nevertheless, the colocalization analysis found no evidence of any shared causal variants underlying the vitamin C–anxiety association, suggesting that the observed link is neither driven by the same variant or set of variants, nor obscured by linkage disequilibrium. Biological mechanisms underlying this relationship may be mediated through a combination of pathways, including its impact on oxidative stress, inflammation, and neurotransmission (Moritz et al. 2020). Future research should aim to clarify the specific mechanisms involved, such as immune modulation, and may open an avenue for a low‐cost intervention for anxiety disorders.

On the other hand, there is no causal relationship between the genetic signature promoting supplemental use of vitamin D and anxiety disorders. Previous clinical research found vitamin D supplementation did not affect anxiety symptoms (Charoenporn et al. 2024; Tirani et al. 2024), even as a significant negative correlation between vitamin D levels and the severity of anxiety was reported (Domacassé et al. 2024; Abdul‐Razzak et al. 2024). Both ours and another recent MR study on vitamin D and depression revealed no substantial genetic correlation (Lin et al. 2024), thus, pointing at distinct differences in the nutritional support strategies aimed at preventing the development of anxiety. The inconsistency in the results regarding the role of vitamin D in mood disorders highlights the complexity of these conditions and the need for a more nuanced understanding of the nutritional factors involved.

For the supplementation with individual vitamins A, B, B6, B12, and E, the multivitamins as a group, and folate, a totality of previous observational and clinical studies that mined for their association with anxiety symptoms remained inconsistent (Pengpid and Peltzer 2024; Nguyen 2022; Borges‐Vieira and Cardoso 2023; Lee et al. 2022; Field et al. 2022). A recent MR study examined the potential causal roles of folate, vitamin B6, and vitamin B12 in anxiety disorders, but only serum vitamin B12 showed a suggestive causal link to disorder risk (Hu et al. 2024). In the present study, we leveraged GWAS summary statistics of substantially larger sample sizes to interrogate a broader panel of vitamins—namely vitamins A, B‐complex (B1, B6, B12), and E—for potential causal effects on anxiety disorders. None of the examined vitamins yielded a statistically significant genetic association; only vitamin E supplementation exhibited a modest directional trend toward causality. Despite the discordant findings, these studies collectively point to a potential link between vitamin status and anxiety disorders. Future research should place greater emphasis on the role and mechanisms of the vitamins in neuropsychiatric conditions.

As for minerals, the MR findings revealed a significant causal association of a lack of supplemental magnesium and selenium with the anxiety phenotype, a marginal trend toward the significance of said correlation with additional iron, but no relationship with supplements containing calcium and zinc. These results suggest that certain micronutrients may play a role in the etiology of anxiety disorders, potentially related to their involvement, either in conjunction with vitamins or independently, in the various biological pathways and neurotransmitter functions associated with anxiety disorders. For instance, some research stresses the involvement of magnesium and selenium in the synthesis and regulation of hormones in the hypothalamic–pituitary–adrenal (HPA) axis and the thyroid axis (Sartori et al. 2012; Sun et al. 2023), while other research points to iron as an element influencing serotonin and dopamine levels in a vitamin E‐dependent manner (Velez Pardo et al. 1995). At least some existing studies have found that supplementing with magnesium, selenium, and iron can be beneficial in alleviating anxiety symptoms (Rawji et al. 2024; Wang et al. 2023). However, a prospective cohort study conducted in China found that the incidence of anxiety increases proportionally to iron and selenium exposure (Zhou et al. 2024). Future observational and interventional studies are warranted to replicate these findings and to clarify the mechanisms through which these minerals may influence anxiety.

Several limitations of our research should be mentioned. Firstly, the datasets utilized in our study did not account for the details concerning the supplementation of exogenous vitamins among study subjects, which could potentially influence the relationships between exposures and outcomes. Secondly, the sensitivity to vitamins may vary by age and sex, yet the available GWAS data did not allow us to stratify our analysis according to these demographic factors. Thirdly, the GWAS statistics utilized in this study were derived from European populations, which limit the generalizability of our findings to other ethnic groups. Lastly, the findings derived from MR analysis should be interpreted cautiously because they did not withstand correction for multiple testing. Therefore, large‐scale GWAS in ancestrally diverse populations, coupled with well‐powered replication studies, are now warranted to clarify the potential contribution of micronutrient supplementation to anxiety disorders.

## 5 Conclusions

In summary, our study provides genetic evidence implicating the potential beneficial effect of vitamin C, magnesium, and selenium intake on the susceptibility to anxiety disorders, suggesting that exogenous supplementation of vitamin C, magnesium, and selenium may mitigate the onset and progression of anxiety.

## Author Contributions

**Xinyu Fang:** writing – original draft, formal analysis. **Qian Zhao:** writing – original draft, methodology. **Peizi Liu:** writing – original draft, formal analysis. **Ancha Baranova:** writing – original draft, methodology. **Hongbao Cao:** writing – original draft. **Xiangrong Zhang:** writing – review and editing, supervision. **Fuquan Zhang:** writing – review and editing, resources, conceptualization.

## Ethics Statement

The authors have nothing to report.

## Consent

The authors have nothing to report.

## Conflicts of Interest

The authors declare no conflicts of interest.

## Supporting information


**Figure S1:** Scatter plot of causal effects of micronutrients intake on anxiety disorder. Scatter plots showing Mendelian randomization (MR) analysis of the effect of genetic variants (SNPs) associated with different micronutrients intake on anxiety disorder. Each panel (A–K) represents the effect of a specific micronutrient intake on anxiety disorder, with SNP effects plotted on the *x*‐axis and corresponding anxiety effects on the *y*‐axis. The three MR methods used are Inverse‐Variance Weighted (IVW, blue), Weighted Median (WM, green), and MR‐Egger (black), with their regression lines overlaid. Error bars indicate the standard error of SNP effects.

**Table S1:** Heterogeneity and horizontal pleiotropy test for these 14 micronutrients.
**Table S2:** Colocalization analysis between vitamin C, magnesium, selenium, and anxiety.

## Data Availability

All data generated or analyzed during this study is included in this published article and its Supporting Information files.
