# Supplementary material for: Causal Influences of Micronutrients on Anxiety: Insights From an Observational and Mendelian Randomization Analysis
Source: Food Sci Nutr. 2025 Nov 5;13(11):e71166. doi: 10.1002/fsn3.71166 (PMC12588952; doi:10.1002/fsn3.71166)

**Supplementary Figure 1. Scatter plot of causal effects of micronutrients intake on anxiety disorder.** Scatter plots showing Mendelian randomization (MR) analysis of the effect of genetic variants (SNPs) associated with different micronutrients intake on anxiety disorder. Each panel (A-K) represents the effect of a specific micronutrient intake on anxiety disorder, with SNP effects plotted on the x-axis and corresponding anxiety effects on the y-axis. The three MR methods used are Inverse-Variance Weighted (IVW, blue), Weighted Median (WM, green), and MR-Egger (black), with their regression lines overlaid. Error bars indicate the standard error of SNP effects.


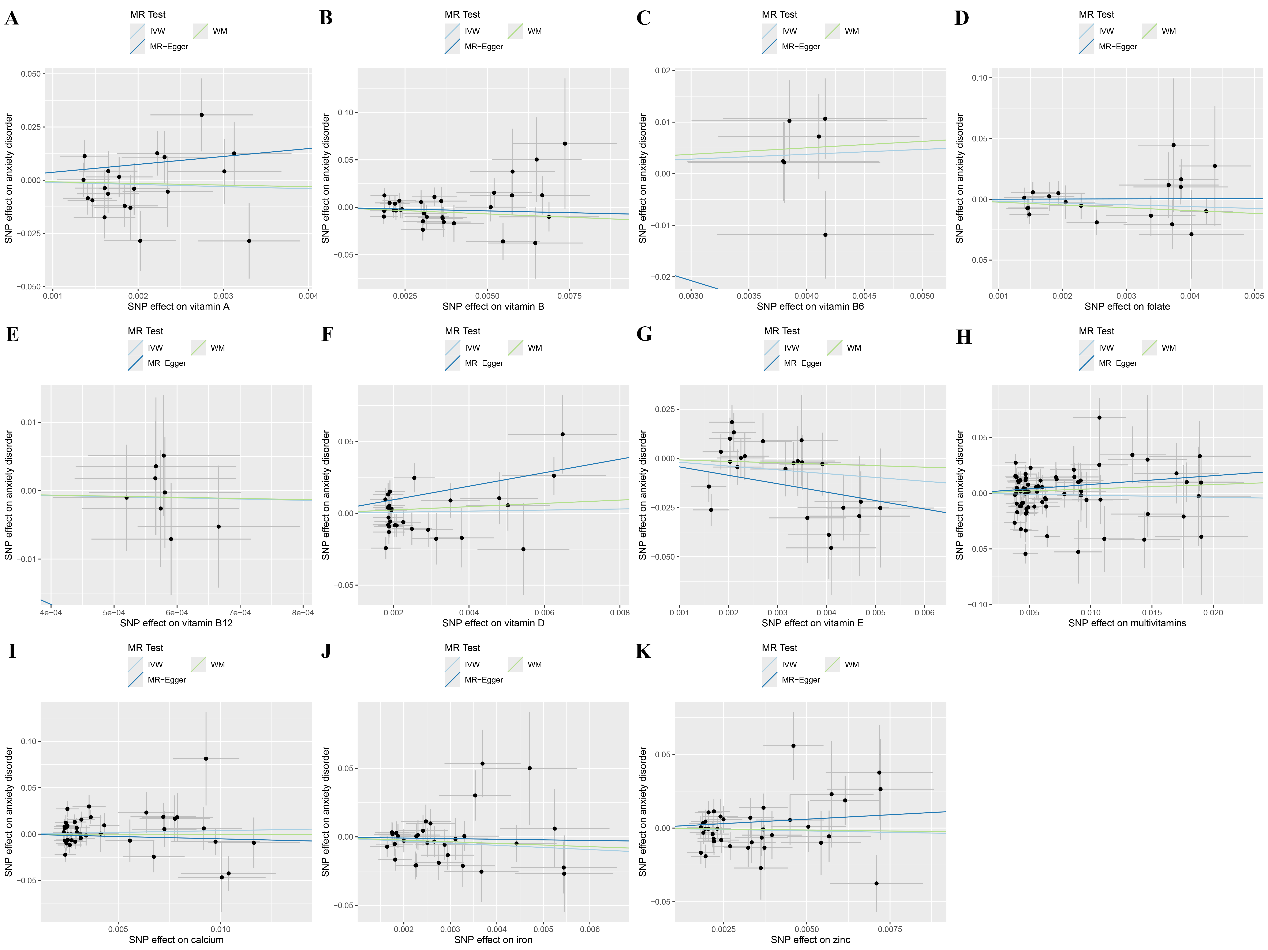

Supplement: Supplementary file 1 — Figure S1: Scatter plot of causal effects of micronutrients intake on anxiety disorder. Scatter plots showing Mendelian randomization (MR) analysis of the effect of genetic variants (SNPs) associated with different micronutrients intake on anxiety disorder. Each panel (A–K) represents the effect of a specific micronutrient intake on anxiety disorder, with SNP effects plotted on the x‐axis and corresponding anxiety effects on the y‐axis. The three MR methods used are Inverse‐Variance Weighted (IVW, blue), Weighted Median (WM, green), and MR‐Egger (black), with their regression lines overlaid. Error bars indicate the standard error of SNP effects. [file FSN3-13-e71166-s001.docx]
